# Comparison between Wrapping Dice Cartilage with Temporal Fascia and Wrapping in Alloderm for Dorsal Nasal Augmentation

**DOI:** 10.29252/wjps.9.2.160

**Published:** 2020-05

**Authors:** Mohammad Bagher Heidari, Mehdi Rasti, Sedigheh Nadri, Arian Roozbehani, Afshin Farhang Fallah, Hormoz Mahmoudvand

**Affiliations:** 1 Department of General Surgery, School of Medicine, Kermanshah University of Medical Sciences, Kermanshah, Iran;; 2 Department of Surgery, Medical School, Isfahan University of Medical Sciences, Isfahan, Iran;; 3 Department of Surgery, School of Medicine, Lorestan University of Medical Sciences, Khorramabad, Iran;; 4 Student Research Committee, Lorestan University of Medical Sciences, Khorramabad, Iran

**Keywords:** Dorsal, Diced cartilage, Alloderm, Fascia

## Abstract

**BACKGROUND:**

Dorsal augmentation of the nose for aesthetic and reconstructive purposes is an important issue in rhinoplasty surgery. This study aimed to compare the two methods of dice cartilage wrapping for dorsal augmentation of the nose including temporalis fascia and alloderm.

**METHODS:**

In a clinical trial study, 50 patients who needed to augment the nasal dorsum, were enrolled and randomly allocated to two equal groups. In the first group, diced cartilage graft of the patient was wrapped in temporalis fascia and in the second group, a thin sheet of alloderm was used for this purpose. After one year follow up, satisfaction of patients and the expert panel were compared in two groups. Also mean increase in dorsal height was measured and compared in two groups.

**RESULTS:**

The mean increase of dorsal nasal height one year after surgery in the alloderm and temporalis fascia was 3.13±0.49 and 3.42±0.33, respectively and in the fascia group was significantly higher (*p*=0.02). The mean of patients’ satisfaction in the two groups of alloderm and temporal fascia groups was 7.48±0.92 and 8.04±0.89, respectively (*p*=0.03). The mean satisfaction of expert panel in the two methods was 7.56±0.81 and 7.7±0.63, respectively (*p*=0.5).

**CONCLUSION:**

The use of temporal fascia for covering the diced cartilage in augmentation of nasal dorsum had better results than the alloderm. Patients satisfaction and mean dorsal height was higher in temporal fascia group.

## INTRODUCTION

Cartilage grafts have formed an integral part of various plastic surgery fields, especially rhinoplasty, and surgeons can exploit different sources including nasal septum, ear concha and rib cartilage.^1^^,^^2^ In contrast to bone that has the capability for extensive regeneration, cartilage lacks internal vascular network and thus, has a limited capability for the reconstruction and regeneration.^3^^,^^4^ Therefore, damage to cartilage often causes scar and permanent loss of structure and function.^5^

The costal cartilage blocks are a rich source of cartilage grafts, but they have some disadvantages including the problems due to the deformity and prominence of cartilage edges.^6^ In a technique, the autogenous cartilages were initially diced into 0.5-1 mm parts in 1 mL of patient blood, but in this method, the use of temporal fascia was proposed due to cartilage absorption. The basic point in these methods is the concerns about the cartilage absorption rate in the post-operative period.^7^^-^^9^

Different studies have been conducted on the methods of preventing from the absorption of cartilage graft and the results have been compared. From the physiologic point of view, it seems that wrapping the diced cartilage with any covering can act as a barrier against the release of substances for the nutrition and reduce the chondrocyte survival rate. Temporal fascia is the autogenous tissue of the patient and thus, has a higher biocompatibility, but it provides a thin covering and is technically difficult to work with. In addition, obtaining the graft product requires spending more time and the morbidity of the area under the graft.

Alloderm is the cadaveric dermis which is acellular and freeze-dried by processing and thus, is nonimmunogenic. It is commercially available in different sizes and thicknesses. Alloderm is flexible and easy to work with. The graft quality resolves the longer operation time and morbidity of the recipient. Besides, the above acellular dermal matrix is integrated into the patient tissue with the mechanism of vascular and cellular regeneration. It is rarely accompanied by the protrusion and shift to the sides.^10^ This study was conducted aiming to compare the effect of using the temporal fascia and alloderm for wrapping the diced cartilage in the dorsal nasal reconstruction.

## MATERIALS AND METHODS

This study was a clinical trial which was conducted in medical centers in Isfahan, Iran during 2016 and 2017. The statistical population included the patients who needed the increased volume and height of nasal dorsum and had congenital, traumatic and iatrogenic (secondary cases) defects due to various causes. The inclusion criteria included the patients candidate for the increased volume and height of nasal dorsum, patients consent to participate in the study, and patients without metabolic and underlying diseases such as diabetes and autoimmune diseases, and absence of any smoking habit. 

Also, the exclusion criteria were no subsequent patient referral, cancellation of operation due to various causes and occurrence of unexpected incidents and trauma leading to nasal injury during the follow-up period. The sample size required in the study was estimated using the formula for estimating the sample size to compare the two means considering the confidence level of 95%, power of test of 80%, standard deviation of dorsum height which to be about 1 mm^6^ and minimum significant difference between the two patients and healthy groups which was considered equal to 0.8 for 25 patients in each group.

After obtaining the permission from the institution ethics committee, 50 patients who were candidate for the nasal dorsum reconstruction and met the inclusion criteria were randomly divided into two groups of 25 patients according to the time of admission to the hospital. In this way, the first patient was randomly assigned to one of the two groups and the subsequent patients were divided into two groups in a successive and alternate manner according to the admission time until the sample size reached the required number in each group. To prevent the confounding results, it was tried to match the patients into two groups according to age and gender distribution. Due to the special intervention conditions, the blinding was not possible in this study. 

In the first group, the temporal fascia of the patients was used to wrap the diced cartilage and was implanted in the nosal dorsum, and a thin alloderm was used in the second group. The surgical operation was performed in all patients under general anesthesia and the operation conditions were similar in all patients too. The standard nasal photography was performed before the operation, during the subsequent visits and follow-up period one year after the operation. The correct photographic principles were observed for the comparison including the amount of light, distance, background, and use of identical camera. 

Five criteria were used to compare the findings including mean difference of dorsum height before and 1 year after the nasal dorsum operation, patient’s satisfaction, expert panel’s satisfaction, postoperative dorsal irregularity, and need for revision. The patient’s and expert panel’s satisfaction was assessed using a 5-point Likert scale including completely satisfied, satisfied, neutral, dissatisfied and completely dissatisfied. The expert panel consisted of 5 surgeons subspecialist on plastic surgery. After collecting data with SPSS software (Version 25, Chicago, IL, USA), data were analyzed by Chi-square, T-test and Mann-Whitney test to compare the regularity of dorsum and need for reconstruction. 

## RESULTS

Table 1 shows the distribution of demographic and general variables of all patients in two groups under dorsum reconstruction with alloderm and temporal fascia graft. According to the results, the age and gender distribution and body mass index (BMI) were not significantly different between the two groups (*p*=0.83, *p*=0.57 and *p*=0.98, respectively). The patient’s and expert panel’s satisfaction with the outcomes of surgery during the one year after the surgery showed that the patients under temporal fascia reconstruction were more satisfied, as the mean satisfaction scores in alloderm and fascia groups were 7.48±0.92 and 8.04±0.89, respectively (*p*=0.033).

**Table 1:** Distribution of demographic and general variables in both groups

**Variable**		**Group**		***p*** ** value**
		**Alloderm**	**Temporal fascia**	
Age Average (year)		32.6±7.24	32.2±5.82	0.83
Sex	Male	13 (52)	11 (44)	0.57
	Female	12 (45)	14 (56)	
Average BMI		25.47±3.71	25.44±3.61	0.98

But the mean satisfaction of expert panel with the outcomes of surgery was not significantly different between the two treatment methods (*p*=0.5). The mean scores of panel expert satisfaction in the both alloderm and fascia groups were 7.56±0.81 and 7.7±0.63, respectively. The mean differences in height of nasal dorsum during one year after the surgery in the both groups under treatment with alloderm and temporal fascia were 3.13±0.49 and 3.42±0.3, respectively, and were significantly higher in the group under treatment with temporal fascia.

According to the examinations, the dorsum regularity during the 1 year post-operation was excellent in 9 patients in alloderm group and in 11 patients of fascia group (36% vs. 44%). Also, it was evaluated as very good in 11 patients of alloderm group and in 12 patients of fascia group and was good in 5 patients of alloderm group and in 2 patients of fascia group, but the difference between the both groups was not significant (*p*=0.47) (Table 2). 

**Table 2:** Mean and standard deviation of dorsum height and expert panel opinion about regularity of dorsum in both groups

**Variable**		**Group**		***p*** ** value**
		**Alloderm**	**Temporal fascia**	
Mean deviation of dorsum height		3.13±0.49	3.42±0.3	0.01
Regularity of dorsum	Excellent	9 (36)	11 (44)	0.47
	Very Good	11 (44)	12 (48)	
	Good	5 (20)	2 (8)	
	Bad	0 (0)	0 (0)	
Need for reconstruction	None	8 (32)	13 (52)	0.05
	Deniable (+1)	9 (36)	11 (44)	
	Better to operate (+2)	7 (28)	1 (4)	
	Obvious necessity for reoperation (+3)	1 (4)	0 (0)	

According to the results, the nasal dorsum did not need reconstruction in 8 patients of alloderm group and in 13 patients of fascia group (32% vs. 52%), but the difference between both groups was not significant (*p*=0.057). Table 3 shows the frequency distribution of patients’ and expert panel’s satisfaction, dorsum regularity, dorsum height difference and not needing any reconstruction in terms of age, gender, cause of injury, BMI, and treatment method. According to the results, the dorsum regularity was significantly different in terms of age and was higher in the age group under 30 (*p*=0.024). 

**Table 3:** Frequency distribution of patients’ and expert panel’s satisfaction, dorsum regularity, dorsum height difference, and needing/not needing for reconstruction in terms of age, gender, cause of injury, BMI, and treatment method

**Variable**		**Patient’s satisfaction**		**Expert panel’s satisfaction**		**Regularity of dorsum**				**Deviation of dorsum height**		**Not need of for reconstruction**	
		**Mean**	***p*** ** value**	**Mean**	***p*** ** value**	**Excellent**	**Very Good**	**Good**	***p*** ** value**	**Mean**	***p*** ** value**	**None**	***p *** **value**
Age	<30	8±0.9	0.18	7.67±0.82	0.79	11 (61.1)	9 (38.9)	0 (0)	0.02	3.11±0.57	0.04	12 (66.7)	0.052
	≤30	7.63±0.94		7.61±0.67		9 (28.1)	16 (50)	7 (21.9)		3.37±0.3		9 (28.1)	
Sex	Male	7.63±1.1	0.33	7.6±0.86	0.81	10 (41.7)	9 (37.5)	5 (20.8)	0.34	3.27±0.59	0.96	10 (41.7)	0.96
	Female	7.88±0.82		7.65±0.58		10 (38.5)	14 (53.8)	2 (7.7)		3.28±0.22		11 (42.3)	
BMI	Normal	7.75±0.93	0.93	7.66±0.73	0.94	12 (42.9)	11 (39.3)	5 (17.9)	0.76	3.33±0.3	0.06	11 (39.3)	0.47
	Over weight	7.71±1.07		7.61±0.68		6 (42.9)	7 (50)	1 (7.1)		3.06±0.63		8 (57.1)	
	Obese	7.88±0.83		7.56±0.82		2 (25)	5 (62.5)	1 (12.5)		3.46±0.29		2 (25)	
Therapy Method	Alloderm	7.48±0.92	0.033	7.56±0.81	0.5	9 (36)	11 (44)	5 (20)	0.55	3.13±0.49	0.01	8 (32)	0.057
	Fascia	8.04±0.89		7.7±0.63		11 (44)	12 (48)	2 (8)		3.42±0.3		13 (52)	

There was also a significant difference in the dorsum height in the age group under 30 years (*p*=0.044). According to the results, the distribution of other factors was not significantly different in terms of gender and BMI, and, as mentioned before, the patient’s satisfaction and the difference in height of dorsum were significantly different based on the treatment method. 

## DISCUSSION

The defect of nasal dorsum is one of the important problems regarding the aesthetic and functional aspects, and thus, it is essential to be reconstructed. Temporal fascia and alloderm are two methods commonly used for the regeneration and reconstruction of nasal dorsum, but there is a disagreement about the priority of each method. Therefore, the present paper aimed to evaluate the results from th application of temporal fascia in comparison with alloderm for wrapping the diced cartilage in the nasal dorsal augmentation.^11^

The satisfaction of patients and expert panel with the treatment outcomes was considered as one of the major elements of recovery. The results showed that the satisfaction of patients with the temporal fascia method was significantly higher than that with the alloderm method. In one study, the impact of using alloderm and temporal fascia on the nasal dorsum reconstruction was evaluated, and the recovery and satisfaction of patients in the temporal fascia group were more favorable during the 15-month follow-up period.^11^

In another study conducted during 2009-2012, a total of 175 patients underwent nasal dorsum reconstruction surgery and were followed up for an average of 1.5 years, and the satisfaction with the outcomes of surgery in the group treated by fascia was 81%, while according to the surgical specialists, the nosal dorsum reconstruction was not satisfactory in 19% of patients.^12^ However, in a study on 83 patients needing nasal dorsum reconstruction with alloderm, this method was found to be successful in the nasal dorsum reconstruction.^13^

When the diced cartilages were wrapped by surgical, a high absorption rate was noted and hence, used the temporal fascia instead.^2^^,^^9^ Surgicel is an oxidized cellulose polymer that is used as a substance for homeostasis and its application as the covering of diced cartilage is associated with inflammation and high absorption of cartilage. The use of alloderm for wrapping the diced cartilage was studied, and good results were presented by this method.^14^

In other studies, on the other hand, the results from the application of alloderm have been conflicting and have mostly mentioned the unreliability and high absorption rate of alloderm. The present study compared the alloderm with temporal fascia for wrapping the diced cartilage for the nasal dorsal augmentation, and the statistical results indicated the superiority of fascia over alloderm. The possible justification for the poorer results of using the alloderm is probably because of the more inflammatory reaction relative to the temporal fascia that is an autogenous tissue, and eventually led to greater absorption and irregularity following the use of alloderm for wrapping the diced cartilage.^15^^-^^18^

Further, the penetration and permeability rates can be considered as the possible causes. Therefore, although the use of temporal fascia causes more time spent for the surgery and is associated with the morbidity of the donor area, the application results are more reliable, and hence, it is recommended to be preferably used. However, the results of various studies have shown that a number of patients undergoing rhinoplasty required reoperation to resolve the anatomic and aesthetic drawbacks for a variety of reasons, and in a significant number of patients, the nasal dorsum reconstruction was the only way for the nose regeneration.^15^^-^^18^

Consequently, using the diced cartilage wrapped by temporal fascia and alloderm are two commonly used methods, and the effect of both methods on the nasal dorsum resconstruction has been reported to be favorable, but the results of our study showed that using the temporal fascia covering was associated with the greater patient’s satisfaction and more favorable height of the nasal dorsum. The use of diced cartilage greatly enhances the range of agmentation with autologous cartilage and is currently the preferred method for many surgeons.^15^^-^^18^

Therefore, it seems that the use of fascia is more desirable than alloderm derived from the cadaveric dermis, because it is from its own tissue, is more biocompatible with the patient, and is less likely to be absorbed. However, given the limitations of this study including the small sample size and lack of similar studies, it is recommended to conduct further studies in this area. The results of our study showed that using the diced cartilage covering was more favorable relative to the use of alloderm covering and was associated with the greater patient’s satisfaction and more favorable recovery of the nasal dorsum height. 
